# MegaPX: fast and space-efficient peptide assignment method using IBF-based multi-indexing

**DOI:** 10.1093/bioinformatics/btag134

**Published:** 2026-05-07

**Authors:** Ahmad Lutfi, Tanja Holstein, Sandro Andreotti, Thilo Muth

**Affiliations:** Data Competence Center MF2, Robert Koch Institute, Berlin, 13353, Germany; VIB-UGent Center for Medical Biotechnology, VIB, Gent, 9052, Belgium; BioOrganic Mass Spectrometry Laboratory (LSMBO), IPHC UMR 7178, University of Strasbourg, CNRS, Strasbourg, 67000, France; Infrastructure Nationale de Protéomique ProFI, UAR2048, Strasbourg, 67087, France; Department of Mathematics and Computer Science, Institute of Computer Science, Freie Universität Berlin, Berlin, 14195, Germany; Data Competence Center MF2, Robert Koch Institute, Berlin, 13353, Germany

## Abstract

**Motivation:**

A central problem for metaproteomic analysis is the often-unknown taxonomic composition of the analyzed microbiomes. Using a database search, the standard approach requires prior knowledge of which proteins and taxa to include in the protein reference database or to use tailored metagenome-derived databases, which are expensive and error-prone in their generation. A possible strategy to circumvent this database search issue is *de novo* sequencing, where peptide sequences are directly identified from mass spectra. However, these sequences must still be mapped back to potentially extensive databases. Here, alignment-based approaches enable robust and precise results, with the potential drawback of high memory usage and long run times.

**Results:**

We present MegaPX, a software for rapidly classifying *de novo* peptide sequences against large protein databases. MegaPX implemented as a C++-based tool, uses an alignment-free, *k*-mer approach as a taxonomic classification method with the possibility of generating mutated reference databases for error-tolerant searching. It uses various algorithms, including interleaved Bloom filters, to efficiently compute approximate membership queries, ensuring fast processing times while querying and indexing large databases in a multi-indexing fashion. We demonstrate the potential of MegaPX by analyzing different samples, including metaproteomics, against extensive reference databases, highlighting its use as a fast screening tool.

## Introduction

Proteomics is a powerful and rapidly evolving approach to study the complete set of proteins expressed by organisms, tissues, or cells. It reveals detailed insights into protein expression and modifications under different conditions, supporting research in medicine, microbiology, pharmacy, industry, and drug development (KhalKhal et al. 2019).

Metaproteomics extends proteomics to large-scale protein analysis in microbial communities, revealing microbial diversity, metabolism, and host–microbe interactions. Unlike traditional proteomics, it examines complex environments, aiding research in microbiomes, environmental microbiology, and infectious disease diagnostics (Kleiner 2019). This field often includes the quantitative profiling of proteins, interactions, and modifications using mass spectrometry (MS)-based analytics (Sinha and Mann 2020).

Among MS methods, liquid chromatography–tandem mass spectrometry (LC–MS/MS) is a high-performance analytical tool increasingly employed in a variety of research and diagnostic applications. The most commonly used method in protein analysis is the bottom-up approach (El Kennani et al. 2018) (Supplementary Material Fig. S1). This method begins with extracting proteins from the target tissue or cell, followed by enzymatic cleavage using a specific enzyme, typically trypsin, which efficiently and precisely cleaves proteins into shorter peptides. These peptides, usually ranging from 5 to 30 amino acids, are separated by liquid chromatography. In tandem mass spectrometry (MS/MS), peptides are first ionized, generating so-called precursor ions, which are then introduced into the mass spectrometer. The MS1 spectrum records the mass-to-charge ratios (*m/z*) of these precursor ions, providing insights into their molecular composition. Selected precursor ions are then fragmented, producing MS2 (or MS/MS) spectra. These fragment spectra are analyzed using computational methods, most commonly database searching or *de novo* peptide sequencing, to identify and quantify peptides in the sample (Wang and Wilson 2013). This process is invaluable for determining protein functions, discovering biomarkers (Karlsson et al. 2020), and supporting drug discovery (Wang et al. 2022).

Database-dependent search engines, such as MS-GF+ (Kim and Pevzner 2014) and Mascot (Brosch et al. 2009) match MS/MS spectra to peptides derived from protein sequence databases. The growing size of protein sequence databases, often containing millions of entries, increases computational demands and impacts statistical validation. Additionally, databases may be incomplete, lacking certain proteins or splice variants. Many peptide sequences are also highly similar due to common sequence motifs, post-translational modifications (PTMs), or housekeeping genes. Furthermore, MS data itself is often noisy, incomplete, or contains overlapping peaks, complicating accurate peptide identification (Verheggen et al. 2016).

In contrast to database searching, *de novo* peptide sequencing identifies peptides directly from MS/MS data without relying on protein sequence databases. Recent advances in machine and deep learning have enabled real-time peptide sequence prediction by training on millions of annotated spectra. This approach is particularly valuable when analyzing samples with unknown or highly variable proteomes, such as cancer cells or viral populations. However, limitations remain due to the lack of comprehensive annotated datasets, restricting the full characterization of viral or cancer-associated proteomes.

Several *de novo* sequencing algorithms have been developed, including SMSNet (Karunratanakul et al. 2019), Novor (Ma 2015), DeepNovo (Tran et al. 2019), PointNovo (Qiao et al. 2021) and Casanovo (Yilmaz et al. 2024). A common challenge these methods face is intrinsic spectrum ambiguity: incomplete or uneven fragment ion coverage leads to characteristic local errors, including single-amino acid substitutions among isobaric residues, short insertions or deletions in sequence tags, neutral-loss-induced mass shifts, and uncertainty at peptide termini, Together, these factors limit full-length accuracy on experimental data (Muth and Renard 2018). Recent comparative assessments indicate that modern deep learning approaches exhibit similar but heterogeneous precision and coverage, with substantial, yet incomplete overlap in correct identifications. This suggests that different tools contribute complementary peptide identifications that can be exploited via consensus strategies or rescoring (van Puyenbroeck et al. 2025).

While *de novo* algorithms excel at identifying peptide sequences from MS/MS spectra, they do not inherently infer the corresponding proteins. As a result, *de novo* sequencing alone is insufficient for full proteome characterization. To bridge this gap, these algorithms must be integrated with sequence analysis methods that map identified peptides to proteins using reference databases. Numerous bioinformatics tools focus on sequence comparison techniques to identify and quantify samples in databases. Comparing sequences with each other plays a crucial role in understanding cellular processes, gene identification, dataset annotation, and pathogen detection (Saeed and Usman 2019). By combining *de novo* sequencing with advanced sequence comparison methods, researchers can enhance the accuracy and depth of proteomic studies, particularly in complex or poorly characterized biological systems. Sequence comparison methods typically use alignment-based approaches that assess similarities based on matches, mismatches, and gaps. These methods are classified as local or global alignments, depending on the application. While alignment-based sequence comparison is accurate for identification, it suffers from quadratic time complexity, as comparisons are performed at the string level and runtime increases exponentially with the number of compared sequences (Rubio-Largo et al. 2017). Modern alignment methods can overcome this quadratic time limit. DIAMOND (Buchfink et al. 2015) aligns a small, pre-selected subset of candidates instead of all sequence pairs. Previous approaches have shown promising results by combining *de novo* sequencing with DIAMOND (Kleikamp et al. 2024). Additionally, there are alignment free methods, that use string matching of *de novo* sequenced peptides (Kleikamp et al. 2021) against Unipept (Vande Moortele et al. 2024).

Alternative approaches based on short, overlapping substrings, called *k*-mers, of length *k* are increasingly used for sequence comparison and identification. While *k*-mer methods have occasionally been applied as tag-based approaches in proteomics (Dasari et al. 2010, Kopczynski et al. 2017), they remain relatively rare in this field. In contrast, *k*-mers are widely used in genomics, for example in Mantis (Pandey et al. 2018) or Raptor (Seiler et al. 2021) often combined with Bloom filters (Bloom 1970). Because of their efficiency, many *k*-mer methods are coupled with approximate string-matching data structures such as quotient filters (Bender et al. 2012), cuckoo filters (Fan et al. 2014), *k*-distance trees (Bentley and Friedman 1979), or other related techniques.

This work introduces *MegaPX*, an innovative tool for peptide assignment that uses the **I**nterleaved **B**loom **F**ilter (IBF) data structure (Hailemariam Dadi et al. 2018) at its core, offering superior performance compared to traditional methods. MegaPX enables highly efficient and memory-friendly querying of extensive protein databases. To demonstrate its performance, we first evaluate MegaPX using a small cancer dataset to assess its accuracy in protein classification. We then extend our analysis to *de novo* peptides from a metaproteome sample by querying an extensive protein database with over 700 million protein sequences. This experiment highlights the feasibility of our tool for execution on standard notebooks with minimal memory consumption. MegaPX’s source code and documentation are available at first mention on GitHub (https://github.com/rki-mf2/MegaPX) under the MIT License, and it can be run on Linux or **W**indows **S**ubsystem for **L**inux (WSL) using conda-based installations.

## Materials and methods

### De novo peptide sequencing

In general, *de novo* peptide sequencing algorithms are designed to directly determine the amino acid sequence of peptides from MS data without referring to a protein sequence database. Many methods are based on machine or deep learning algorithms and attempt to make predictions in real-time with specific parametric configurations. We combined the sequencing results from two different deep learning models: Casanovo (Yilmaz et al. 2024) and SMSNet (Karunratanakul et al. 2019). Both were executed on a Linux machine (Ubuntu 20.04.6 LTS 64-bit) with standard GPU configurations. SMSNet was executed using a previously published *de novo* sequencing pipeline (Beslic et al. 2023). Both deep learning models have been trained on high-resolution MS/MS data from the human proteome using the HCD library from MassIVE Wang et al. (2018), which consists of 1 114 503 annotated peptides. The models were trained with a precursor tolerance of 10 ppm and a fragment mass tolerance of 0.02 Da. Carbamidomethylation of cysteine was set as a fixed modification, whereas oxidation of methionine and deamidation of asparagine and glutamine were set as variable modifications. For the analysis, we opted to use the pre-trained Casanovo weights from the original publication and the SMSNet model from Beslic et al. (2023). This decision was based on their distinct training procedures, ensuring broader coverage of different sequencing scenarios in our combined approach.

To ensure data quality, we filtered *de novo* peptides based on the peptide-level confidence score reported by each model, defined as the average of the amino acid–level confidence scores across the predicted sequence. For all global analyses, we used a default threshold of 70%, which in our datasets corresponds to the high-confidence regime of the score distribution and effectively removes the long tail of low-scoring predictions. This is consistent with typical settings in *de novo* sequencing workflows, where comparable peptide- or amino acid–level confidence cutoffs are used to retain sequences with high expected correctness while controlling the error rate (Tran et al. 2019). At the same time, recent work has shown that *de novo* confidence scores are not perfectly calibrated across spectra and sequence contexts, and that varying the score threshold mainly trades recall for precision rather than defining a hard boundary between correct and incorrect sequences (Mabona et al. 2025, van Puyenbroeck et al. 2025) in regions where we expect a higher rate of genuine sequence variability (e.g. mutated peptides), we therefore relaxed the cutoff to 50%. This more permissive threshold is motivated by the empirical behaviour of the models on our data and by the observations above: lowering the cutoff substantially increases sensitivity to variant peptides while still filtering out most low-confidence predictions. Thus, the 70% threshold is used as a conservative setting for most analyses, whereas the 50% threshold is applied only in mutation-rich contexts where higher recall of true variants is prioritized over maximal specificity.

### Classification task

#### Interleaved bloom filter

To enable efficient simultaneous querying of multiple Bloom filters, Hailemariam Dadi et al. (2018) introduced the **I**nterleaved **B**loom **F**ilter (**IBF**). Unlike conventional approaches that query each filter individually, the IBF combines multiple Bloom filters of equal size *n* into a single interleaved structure using a shared set of hash functions H. Each bit in the IBF is replaced by a sub-bit vector of size *b*, where the *i*-th bit corresponds to bin $B_{i}$, resulting in a total size of b×n. This structure allows all bins to be queried simultaneously, improving efficiency. The false positive rate depends on the size *n*, the number of inserted elements *m*, and the number of hash functions H, and can be calculated as described by Hailemariam Dadi et al. (2018):


(1) $$p={\left(1-{\left(1-\frac{1}{n}\right)}^{H\cdot m}\right)}^{H}$$

In our application, the IBF is used to index *k*-mers derived from peptide sequences across many species-level bins. Because each bin contains a finite set of peptide-derived *k*-mers, the sizing of the underlying Bloom filters must account for the maximum possible number of *k*-mers that any input bin may contribute. This quantity is referred to as *maxKmers* and directly determines the Bloom filter size required to maintain the desired false positive rate.

The optimal size of each Bloom filter (bin) for a given error rate can be calculated using the equation (Ulrich et al. 2022):


(2) $$n=\left\lceil \frac{-1}{\left(r^{\frac{1}{H\times \text{maxKmers}}}-1\right)}\right\rceil ,$$

where $r=1-p^{1/H}$ and *maxKmers* is the maximum number of *k*-mers in the input user bins (input references). This value is computed by estimating the maximum size of *k*-mers sets in the input user bins. Our tool calculates the size of the IBF based on the vectorized representation of the user bins using a default false positive rate of 0.01. Figure S2 in the Supplementary Material shows an example of an IBF structure derived from three different input sequences. In the beginning, each reference sequence is *k*-merized to a length *k*. Subsequently, the generated *k*-mers are encoded, and these encoded *k*-mers are either inserted directly into the filter or mutated versions are generated (processed *k*-mers). The resulting *k*-mers are then hashed using three different hash functions, and the resulting hashes of each reference sequence are inserted into the corresponding Bloom filter. These different BFs are then interleaved into a single IBF. However, it should be noted that memory consumption can increase significantly if the reference sequences are of very different sizes, as all BFs are interleaved. Consequently, the size corresponds to the largest BF, meaning other filters also reserve a lot of memory for empty blocks.

#### Peptide assignment

Following the query membership of peptide sequences from each sample against a pre-built index, we constructed a count matrix *C*. Each row in this matrix represents a peptide, while each column corresponds to the user bin. The entries of the matrix are the *k*-mers’ hits per bin for every query peptide:


(3) $$\begin{array}{c} C=\left(\begin{array}{cccccc} & b_{1} & b_{2} & b_{3} & \cdots & b_{v}\\ p_{1} & c_{11} & c_{12} & c_{13} & \cdots & c_{1v}\\ p_{2} & c_{21} & c_{22} & c_{23} & \cdots & c_{2v}\\ p_{3} & c_{31} & c_{32} & c_{33} & \cdots & c_{3v}\\ \vdots & \vdots & \vdots & \vdots & \ddots & \vdots \\ p_{u} & c_{u1} & c_{u2} & c_{u3} & \cdots & c_{uv} \end{array}\right) \end{array}$$

The count matrix is further processed according to the user-defined assignment threshold *D*, representing the percentage of the peptide *P* (number of *k*-mers) that matches the user bin *B* to assign the peptide to that user bin. Let $x=y\times \frac{D}{100}$, where *y* is the total number of *k*-mers in peptide *P*.


(4) $$L_{uv}=\{\begin{array}{c} 1\;\;\;\text{if}\;C_{uv}\geq x\\ 0\;\;\;\text{if}\;C_{uv}<x \end{array}$$

The lookup matrix is built with bits (1 and 0) and defined as:


(5) $$L=\begin{array}{cccccc} & b_{1} & b_{2} & b_{3} & b_{4} & b_{v}\\ p_{1} & l_{11} & l_{12} & l_{13} & l_{14} & l_{1v}\\ p_{2} & l_{21} & l_{22} & l_{23} & l_{24} & l_{2v}\\ p_{3} & l_{31} & l_{32} & l_{33} & l_{34} & l_{3v}\\ p_{4} & l_{41} & l_{42} & l_{43} & l_{44} & l_{4v}\\ p_{u} & l_{u1} & l_{u2} & l_{u3} & l_{u4} & l_{uv} \end{array}$$

Using the lookup table 50, the assignment score *S* of the set of peptides in a sample to the corresponding user bin *B* can be computed as:


(6) $$S=\frac{\sum_{f=1}^{z}l_{fB}}{z},$$

where *z* is the total number of peptides in the input sample. MegaPX supports querying large databases with errors, enabling an error-tolerant detection of amino acid sequences. Substitution matrices characterize all possible modifications of each amino acid by another and provide essential information for predicting potential changes based on a specific score. The BLOSUM (**BLO**cks **SU**bstitution **M**atrix) series is one of the most widely used substitution matrices. BLOSUM matrices are constructed by clustering aligned sequence segments within conserved blocks based on a specified percentage identity threshold. This process involves analyzing large protein data sets to identify amino acid substitution patterns, which are then used to derive scoring matrices that reflect the likelihood such substitutions occurring in related proteins (Henikoff and Henikoff 1992). BLOSUM62 is derived from sequence blocks with at least 62% identity (Henikoff and Henikoff 1992). In a BLOSUM matrix, higher scores indicate a greater probability that the corresponding amino acid substitution occurs during sequence alignment. We generated all possible mutational variants of each *k*-mer by simulating potential amino acid substitutions, guided by sequence similarity, to identify target proteins even in the presence of mutations. The process begins by dividing the target protein sequence into overlapping *k*-mers. For each *k*-mer, we consider all possible amino acid substitutions based on the BLOSUM62 substitution matrix and a user-defined similarity score threshold. Without optimization, generating mutational variants requires evaluating up to $20^{k}$ combinations, making it computationally expensive. To reduce runtime, we pre-sort the substitution matrix so that, for each amino acid, candidates are ordered by descending similarity score. This prioritizes high-scoring substitutions during variant generation. An early stopping mechanism further improves efficiency: if a substitution score falls below a user-defined threshold, the process terminates early, skipping remaining lower-scoring candidates. This reduces computations when aligning mutated *k*-mers to the reference, enhancing runtime performance. The generated database includes both original and mutated *k*-mers, ensuring that the original protein sequences are also represented in the IBF.

#### Multi-indexing classification

**Figure 1:**
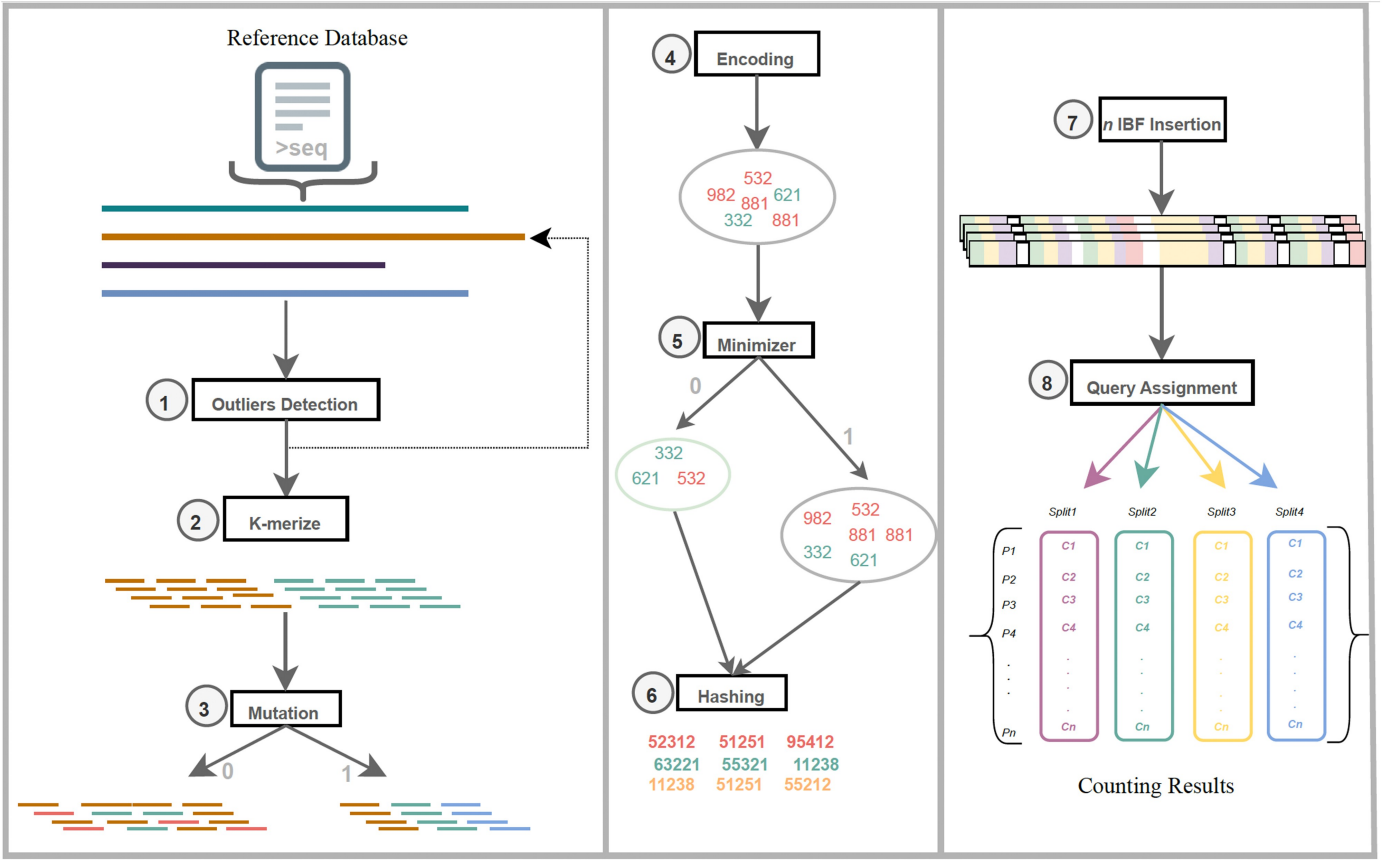
Multi-indexing approach workflow. The user bins are loaded in memory in batches according to the user-defined *M* split size; (1) Removing outliers according to the user-defined black list. (2) *K*-mer computation of each reference sequence in the loaded split. (3) Generating mutation according to user-defined score, if needed (Boolean value 0 or 1). (4) Encoding the *k*-mers to the 64-bit integer representation. (5) Compute minimizers of each *k*-mers set. (6) Hash the encoded values using set of hash functions *H*. (7) Insert the *k*-mers into the target IBF; each IBF has the same size as the split size (the last IBF contains the rest of the sequences). (8) Query assignment of input peptides against the built index. The query and assignment step is repeated until all IBFs are queried without outliers. The different colors represent the different splits/IBFs.

MegaPX uses a multiple indexing strategy based on a divide-and-conquer algorithm to efficiently handle large databases. It partitions *n* user-defined references into batches, generating separate IBFs with equal numbers of user bins (based on the split size). Each IBF is sized according to its content to minimize filter size and false positives. By breaking the problem into smaller sub-tasks and combining the results, this approach reduces both time and memory consumption. While this approach optimizes indexing efficiency, it may lead to slower query times, particularly when the target database contains a high proportion of over-represented sequences. To address this, MegaPX allows the inclusion of an external blacklist file, enabling users to exclude specific references from the indexing process. These references, often considered outliers, are omitted from subsequent searches to improve query performance. This method eliminates the need to load, write, and repeatedly query very large IBFs. Instead, it enables querying smaller IBFs, which can be efficiently managed within standard RAM configurations. The workflow of this approach is illustrated in Fig. 1 (pseudo-code in Supplementary Material Algorithm S1). Additionally, our workflow supports minimizer computation. Minimizers are typically calculated in both the forward and reverse directions of a given sequence, as DNA sequences have palindromic features where the sequence on one strand is the reverse complement of the sequence on the other strand (Miklenic and Svetec 2021). Taking both the forward and reverse strands into account is crucial for applications such as sequence alignment, where similarities can occur on either strand [https://www.cureffi.org/2012/12/19/forward-and-reverse-reads-in-paired-end-sequencing/]. Unlike DNA, protein sequences consist of amino acids and lack a reverse complement, so minimizers are calculated only on the forward strand. As protein sequences are typically shorter, minimizer calculation is included as an optional step. The workflow begins with preparing input datasets, including the reference database and MS/MS spectra (in MGF or converted from RAW). Input protein sequences are *k*-merized to a specified length *k* and inserted into the IBF index. *De novo* peptides are sequenced from spectra, *k*-merized at the same *k*, and searched against the index (Supplementary Material Fig. S3). Finally, taxonomy is assigned based on peptide-to-protein matches, with scoring based on Equation (6).

## Experiments

All applied experiments are explained in the Supplementary Material.

## Results

We conducted three main experiments to evaluate the performance of our tool in searching and classifying peptide sequences within large protein databases. The results are presented in subsections, which focus on error-free searches and error-tolerant searches that incorporate mutation generation. Finally, we assess potential diagnostic applications. A detailed description of the experimental setup is provided in the Supplementary Material.

### Performance evaluation of error-free peptide assignment

All analyses in this section were conducted using the Mix24X metaproteomics dataset (Mappa et al. 2023) which has a defined taxonomic composition. This dataset was used to benchmark MegaPX against alternative methods such as DIAMOND. Both *de novo* peptides (69 656 unique sequences) and Mascot-derived peptides (77 434 unique sequences) were searched against a combined reference database comprising Mix24X target sequences and the NCBI RefSeqBacterial database (O’Leary et al. 2015). Given that IBF searches are approximate, it is essential to demonstrate the tool’s ability to search within highly similar sequences and accurately assign hits.

To further explore this capability, we constructed proteomes from individual proteins, moving from the protein level to the species level. The primary purpose of combining proteins into proteomes was to showcase MegaPX’s capability to accurately assign hits for short peptides within extensive search spaces while utilizing minimal memory usage.

The first search, performed with MegaPX using *de novo* peptides, was completed in 4.68 min, consuming 2.18 GB of memory (Supplementary Material Table S1). In comparison, the second search, using Mascot peptides required 4.8 min and the same memory allocation. Memory usage could potentially be further optimized by reducing split sizes. We assessed the overlap between *de novo* and Mascot-derived peptides using sequence similarity analysis. Only 580 peptides matched exactly, but global alignment revealed a high similarity, with scores ranging from 70% to 98%, reflecting minor length differences and variability from *de novo* sequencing. To improve overlap detection, we applied a *k*-mer approach (k=5), which identified partial matches and shared sub-sequences.

**Figure 2:**
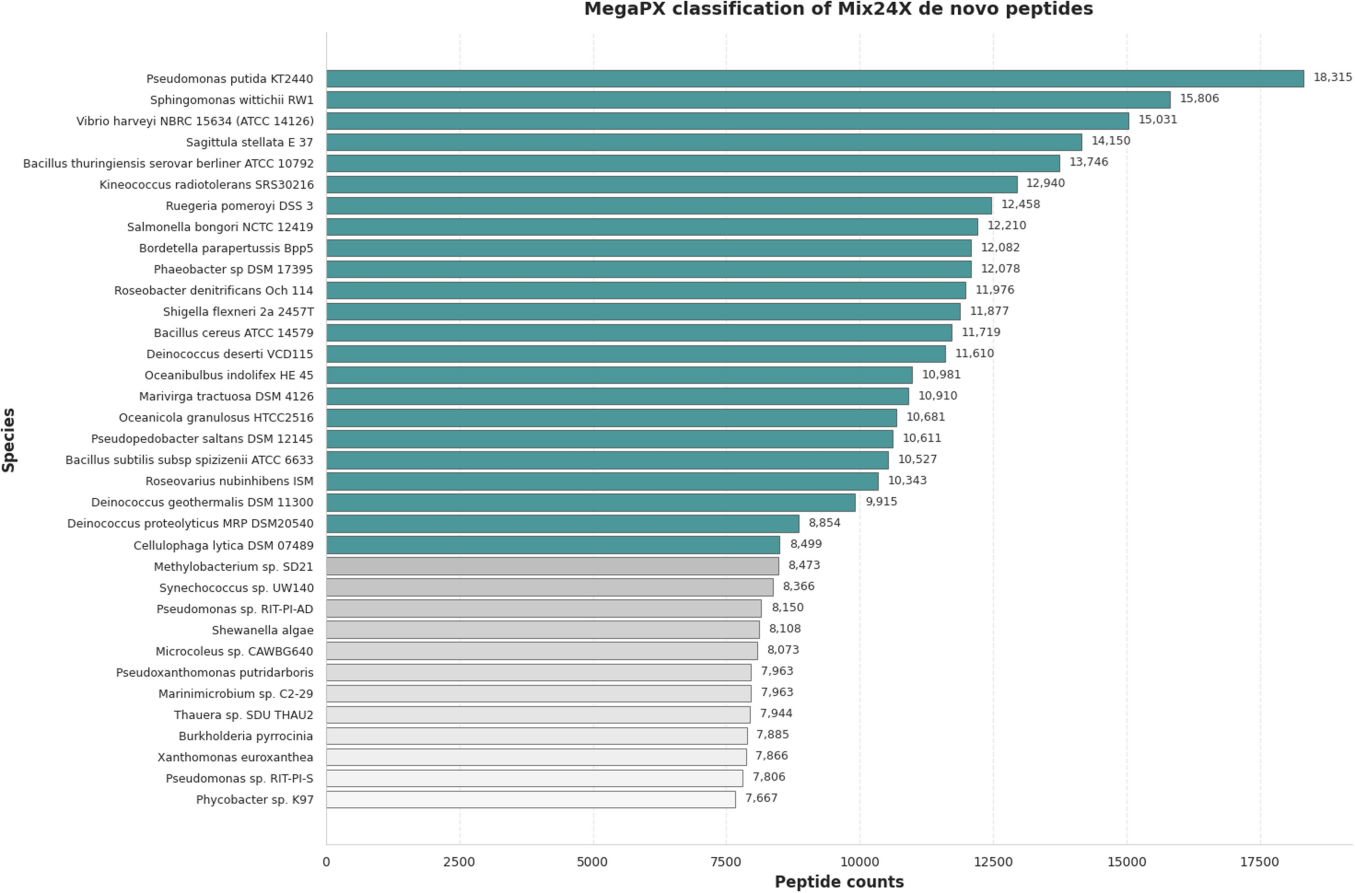
Classification results of *de novo* peptides for the Mix24X sample using MegaPX. The figure shows the top 35 taxa ranked by the number of assigned de novo peptides in the Mix24X sample. Sequences were queried using *k = 5* using a full-coverage assignment criterion (all peptide *k*-mers must be supported by the target). Teal bars correspond to the 23 strains that are known constituents of the Mix24X community, whereas grey bars denote additional taxa from the reference database that are not part of the mixture but share high sequence similarity with Mix24X proteins. MegaPX concentrates the majority of peptide assignments on the expected Mix24X strains (e.g. *Pseudomonas putida* KT2440, *Sphingomonas wittichii* RW1), while the remaining assignments map to closely related taxa, reflecting sequence homology rather than random false-positive classifications.

Figure 2 summarizes the taxonomic classification of *de novo* peptides by MegaPX. MegaPX correctly assigned the dominant share of peptides to the expected Mix24X strains such as *Pseudomonas putida KT2440* and *Sphingomonas wittichii RW1*, which receive the highest peptide counts while a smaller fraction was assigned closely related taxa with high sequence similarity to the Mix24X members. These taxa should therefore not be interpreted as random false positives, but rather as alternative strain-level annotations for shared or conserved peptide sequences. Overall, MegaPX robustly assigns *de novo* peptides quantitatively, with the majority of the signal concentrated on the known community members and additional, biologically plausible assignments to related taxa.

Conversely, *Staphylococcus_carnosus_subsp_carnosus_TM300* was underrepresented, as its shorter proteins resulted in fewer peptide hits. Next, we compared the counts between Mascot-derived and *de novo* peptides (Supplementary Material Fig. S4). The results show that *de novo* peptides achieve a lower number of matches to target strains. However, as expected, database-derived peptides demonstrate higher match precision. This finding highlights an additional application of MegaPX as a screening tool. By using MegaPX to assign preliminary hits, researchers can refine their analyses by focusing on high-confidence matches for database searches, reducing the runtime by narrowing subsequent database searches to a subset of relevant references. Nevertheless, MegaPX, when used with*de novo* peptides, still identifies a substantial number of peptide hits. DIAMOND (version 2.1.9) (Buchfink et al. 2015) was tested on the same dataset for comparison. The database was built in 10.3 s, and the query search on the pre-built database using *blastp* was completed in 93.21 s (Supplementary Material Table S1). DIAMOND aligned three queries and four queries when the search was restricted to target proteins. To address this limitation and allow for a fair comparison—without relying on the approach of concatenating proteins into a single proteome with marker sequences between them—we reconstructed the individual protein sequences from the Mix24X proteomes and performed the search at the protein level rather than the proteome level. We first searched de novo peptides against the Mix24X reference proteins using *DIAMOND*. In this test, the tool reported 6 pairwise alignments, with 4 unique peptide queries successfully aligned using default parameters.

Relaxing the DIAMOND *e*-value threshold (default is 0.001) increased the number of reported hits to 109 at *e* = 0.01 and to 398 at *e* = 10, while runtime remained essentially unchanged. Because the *e*-value reflects the expected number of random matches with equal or better scores, thresholds of e≥1 imply at least one such random hit is expected per search. Consequently, the additional hits that appear at *e*≥0.1 are low-confidence assignments, particularly short peptides where score values are low.

For a more realistic scenario, we performed searches using Mascot-identified peptides (pseudo-ground truth), querying exclusively against target reference proteins with *MegaPX* and *DIAMOND* by using the default parameters. This resulted in a total of 178 520 pairwise alignments, with 40 014 peptide queries successfully aligned. Next we applied recommended settings from the NovoLign method (Kleikamp et al. 2024) tailored for short peptides: *PAM30*, contiguous seed algorithm, 85% identity, 80% query coverage, *max-target-seqs* 50. Using these settings, DIAMOND improved the search results without increasing runtime. Aligned peptides rose from 263 at *e *= 0.001 to 449 at *e* = 0.01, 734 at *e* = 0.1, 1016 at *e* = 1, and 1188 at *e* = 10, with total alignments increasing from 541 to 1974. Despite this improvement, coverage remained very low relative to the input size: 1188 of 234 509 peptides, about 0.51%.

By contrast, MegaPX identified 8077 peptides with at least one reference hit on the same dataset (around 3.45%), representing a 6.8-fold increase over DIAMOND’s best-performing setting. This indicates that relaxing DIAMOND’s *e*-value primarily inflates weak matches, while a *k*-mer presence/absence approach recovers substantially more short-peptide matches at practical runtimes.

Of the 90 616 Mascot peptides, DIAMOND could only align 40 014, representing 44.15% coverage using default parameters. Using NovoLign-style settings increased output to 237 386 alignments and 84 659 aligned peptides (93.4% coverage). MegaPX mapped all peptides (100% coverage) with 59 120 uniquely assigned queries (65.2% of all peptides). These results show that DIAMOND’s default configuration under-calls short peptides, NovoLign-style tuning recovers most sequences, and the *k*-mer-based MegaPX achieves complete coverage with a high fraction of uniquely assigned peptides.

**Figure 3:**
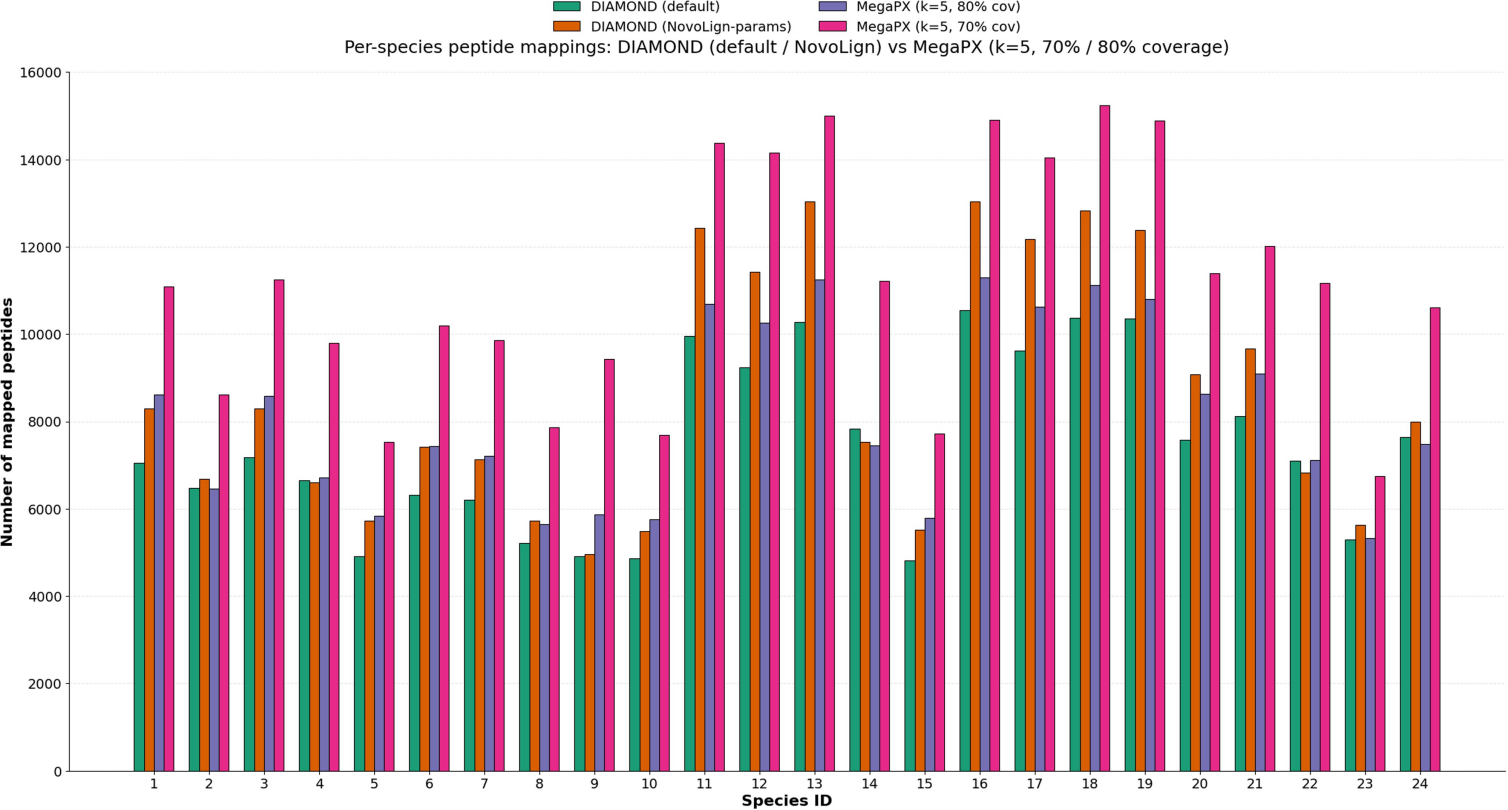
Per-species Mascot peptide mappings against the Mix24X target proteomes. Mascot peptides (*n* = 90 616) were searched exclusively against the 24 Mix24X reference proteomes using DIAMOND with default parameters (green), DIAMOND with NovoLign-style settings (orange; PAM30, 85% identity, *–query-cover* 80), and MegaPX with *k = 5* at 80% (purple) and 70% (pink) peptide coverage thresholds. DIAMOND’s coverage is defined at the amino-acid alignment level (fraction of residues covered by the HSP), whereas MegaPX coverage is defined as the fraction of peptide *k*-mers that pass the counting threshold, so the 80% settings are analogous but not formally equivalent. Bars show the number of mapped peptides per species, highlighting that MegaPX at 80% coverage matches or exceeds DIAMOND with optimized parameters for many targets, and that relaxing the MegaPX threshold to 70% further increases peptide recovery across nearly all Mix24X species. Sample names are in Table S3 in the Supplementary Material.

Figure 3 compares per-species peptide counts obtained with DIAMOND (default parameters), DIAMOND with NovoLign-style settings, and MegaPX with *k = 5* at two thresholds (80% and 70% peptide coverage). It is important to note that *80% coverage* is defined differently in the two tools. In DIAMOND, *–query-cover 80* refers to the fraction of amino-acid positions of the peptide that are included in the reported alignment. In MegaPX, the 0.80 threshold is applied to the fraction of *k*-mers from the peptide that reach the count threshold in a given reference bin; this operates at the *k*-mer level rather than residue-level alignment. Consequently, the two 80% settings are not directly comparable and should be interpreted as analogous but not identical stringency filters. At this nominal 80% level, MegaPX outperforms DIAMOND with optimized parameters for several Mix24X species, while DIAMOND still yields slightly higher peptide counts for others, reflecting differences in how each method scores and filters evidence. When the MegaPX threshold is relaxed to 70% *k*-mer coverage, the number of mapped peptides increases for all targets and generally exceeds both DIAMOND configurations across the panel, illustrating how a *k*-mer–based approach can be tuned to trade coverage against stringency in a way that classical alignment statistics cannot.

We also evaluated PeptideMapper (Kopczynski et al. 2017) in the same context. PeptideMapper and MegaPX are not directly comparable because they address different tasks. PeptideMapper performs *exact substring matching*, reporting a hit only when its full peptide sequence occurs verbatim in a protein, which favors short peptides and rapidly loses sensitivity for longer or error-prone sequences. MegaPX, by contrast, uses *k*-mer-based matching, tolerating substitutions and *de novo* sequence uncertainty. As a result, it can map medium- and long-length peptides that lack exact matches. This explains the different length distributions (PeptideMapper concentrated on very short peptides, MegaPX extends to longer ones) and why totals such as 6123 vs. 8077 reflect different criteria rather than tool performance. Figure S8 in the Supplementary Material provides an illustration of this analysis.

MegaPX demonstrated its scalability by successfully searching the extensive NCBI_nr database, containing 707 million proteins (339 GB). This search, using a standard notebook with five *k*-mer extraction threads, completed in approximately 18 days while consuming only 0.177 GB of RAM (Supplementary Material Table S1). In comparison, DIAMOND faced memory constraints during database building, requiring more than 4 GB of RAM and failing to complete the task. MegaPX’s ability to handle large datasets with minimal resources underscores its utility for large-scale metaproteomics studies. The purpose of searching against NCBI_nr was mainly to demonstrate its potential as a screening tool, capable of identifying top-hit sequences. These hits can then be used to narrow down the database for more focused searches or to guide *de novo* sequencing, optimizing both search efficiency and resource usage; this use case could also contribute to the development of advanced algorithm designs for *de novo* sequencing.

### Error-tolerant classification

#### Lung squamous cell carcinoma

**Figure 4:**
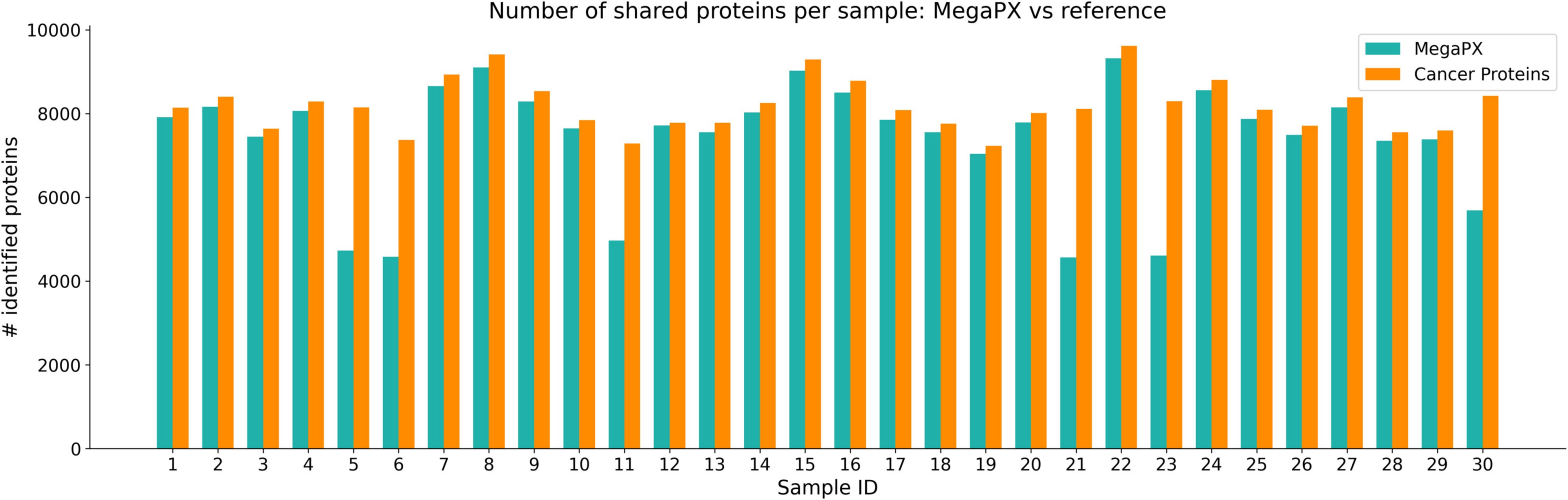
Comparison of the number of proteins identified by MegaPX and the Ground Truth. This figure shows the number of identified proteins per LSCC sample, comparing results from MegaPX with those reported in the original publication based on pseudo-ground truth classifications. To ensure a fair comparison despite differences in reference databases, only shared proteins that were identified by MegaPX and also mentioned in the cancer study were considered. The samples names are in the Supplementary Material Table S4.

To evaluate the ability of MegaPX to identify peptides with high levels of mutations, we conducted a search against a cancer dataset (Satpathy et al. 2021) using the mutation mode based on BLOSUM matrices. This dataset contains numerous modifications, making accurate peptide assignment particularly challenging, as mutations can occur at any amino acid position. Notably, the LSCC datasets include pseudo-ground truth peptides identified using the published pipeline, providing an ideal scenario for analyzing shared peptides between SMSNet and Casanovo *de novo* peptides and the pseudo-ground truth peptides. Figure S5 in the Supplementary Material provides a comparison example of shared peptides between pseudo-ground truth and *de novo* sequenced peptides within two LSCC samples. The *de novo* peptides, derived using SMSNet and Casanovo, have been filtered for duplicates and subjected to a score threshold of 50%. In contrast, pseudo-ground truth peptides may include duplicates and are not restricted by a score cutoff. The visualization underscores the overlap and exclusivity of peptides between datasets. Next, the *de novo* collected peptides are searched against the human reference sequence proteome database (OLeary et al. 2015). All references undergo a mutation to a *k*-mer size of 5 with a mutation score of 25. The original study contains the number and name of the identified proteins for each sample. Figure 4 displays the number of shared proteins identified in each sample. Shared proteins are those identified by MegaPX and are also present in the pseudo-ground truth. MegaPX successfully identified a significant number of proteins from the previous study. However, the pseudo-ground truth results include duplicate proteins and others not present in our reference datasets, as the customized reference database used in the original study was not published. Despite this limitation, MegaPX was able to identify additional proteins from the human proteome reference database. Including these proteins in the results would not allow for a fair comparison with the original publication, as the specific reference database they used was not specified. Therefore, we focused solely on comparing the number of shared peptides/proteins between MegaPX and the published results. Our analysis showed promising results in protein identification scenarios, which facilitates one-to-one identification and peptide assignment of *de novo* peptides with mutation generation. Almost all samples exhibit a high number of matched proteins between cancer and MegaPX results, with a identification recall averaging around 97%, except for four samples (05CPTAC _02, 03CPTAC _01, 03CPTAC _02, 04CPTAC _01) which display protein matching ratios ranging from 57% to 68% (Supplementary Material Table S2). These outliers may indicate discrepancies caused by differences in sample quality, experimental conditions, or limitations in the reference datasets. The overall high percentages for most samples demonstrate the robustness of MegaPX in identifying proteins from the original study. The complete search is performed in about 16 min with less than 0.5 GB memory consumption with 114 IBFs (Supplementary Material Table S1).

#### Evaluation of diagnostic applications

##### Sample search against highly similar proteomes

In order to evaluate diagnostic applications, we used the Mix24X dataset, derived from a lab-assembled mixture sample covering 20 genera, including *Shigella flexneri*, a species closely related to *Escherichia coli* with a high degree of proteome sequence overlap. Our goal was to assess the specificity and sensitivity of the diagnostic method in distinguishing between highly similar species. We used MegaPX to search 69,656 *de novo* sequencing-derived peptides from Mix24X against the *E. coli* and *Shigella flexneri* UniProt protein database (Rolf et al. 2004). The search completed in under 1 min, utilizing only 102 MB of memory in both scenarios. The results shown in Fig. S6 in the Supplementary Material indicate that the combination of MegaPX and *de novo* peptides proves to be highly effective, as it assigns the highest number of peptides to *Shigella flexneri*. This demonstrates a strong use case for MegaPX, particularly in distinguishing between highly similar proteins with high assignment accuracy. Notably, the tool performs consistently well across both high (95%) and low (85%) assignment threshold scenarios, confirming its robustness and reliability in peptide assignment.

##### Species-unique peptides

**Figure 5:**
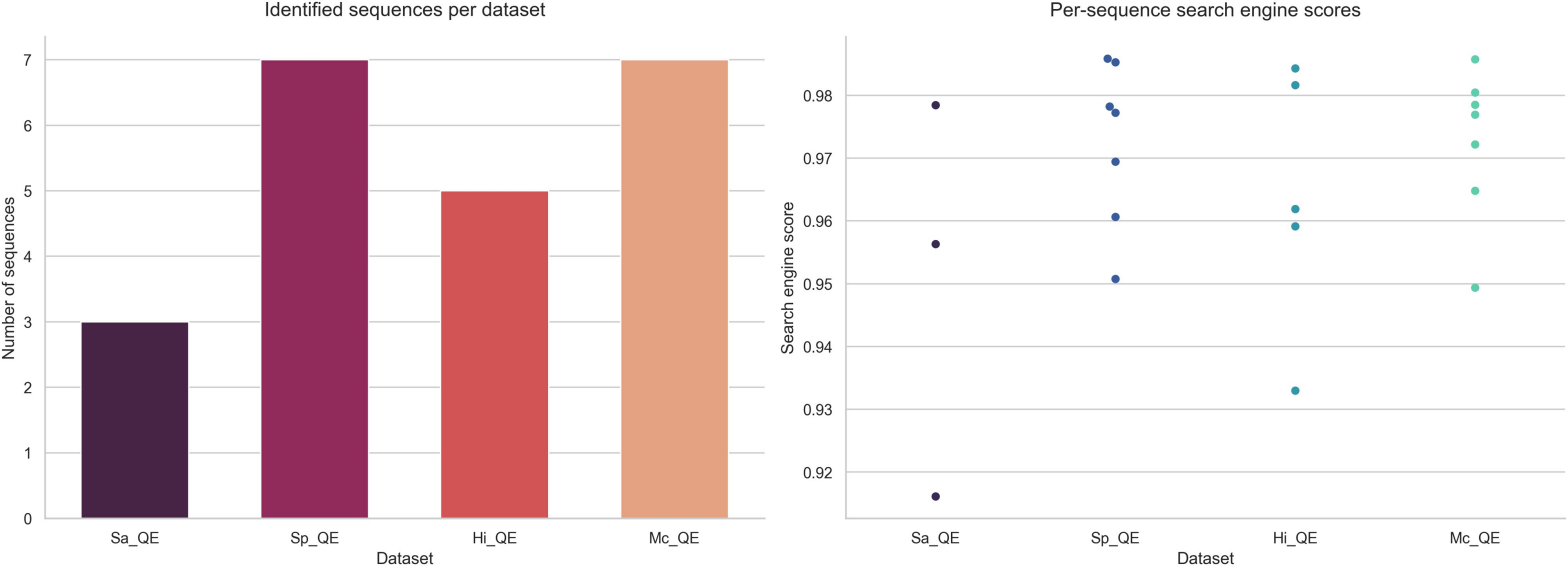
Identified sequences and their corresponding search engine scores across datasets. The bar plot (left) represents the number of sequences identified for each dataset (*Sa_QE, Sp_QE, Hi_QE, Mc_QE*). The swarm plot (right) displays individual search engine scores for all identified sequences, allowing direct visualization of the score distribution and variability per dataset. *Sa_QE* corresponds to *Staphylococcus aureus*, *Sp_QE* to *Streptococcus pneumoniae*, *Hi_QE* to *Haemophilus influenzae*, and *Mc_QE* to *Moraxella catarrhalis*.

To evaluate Casanovo’s ability to identify species-unique peptide sequences in a pathogenic scenario, we applied our approach to datasets of bacterial respiratory pathogens (Karlsson et al. 2020), including *Streptococcus pneumoniae*, *Haemophilus influenzae*, *Moraxella catarrhalis*, and *Staphylococcus aureus*. This experiment builds on the work of [Kar + 20b], which identified promising candidate peptides for these species. Figure 5 presents the number of sequences identified per dataset, along with their corresponding search engine scores from Casanovo. Using Casanovo, we successfully *de novo* sequenced 28 species-unique peptides as exact matches, demonstrating the high precision in peptide identification. Across all datasets, sequence scores were consistently high (>80%), ensuring confidence in the obtained results.

The distribution of search engine scores further confirms that the majority of identified peptides were assigned high confidence values. In addition to exact matches, Casanovo identified numerous partial sequence matches to the original peptides (e.g. NVATDANHSYTSR and **AN**NVATDANHSYTSR, AVVYNNEGTK and AVVYNNEGTK**VELGGR**). However, we did not consider these partial sequences, as our primary objective was to demonstrate the capability of Casanovo for exact peptide sequencing and to validate the diagnostic applicability of MegaPX for pathogen classification. To assess *de novo* sequencing accuracy and consistency at the amino acid level, we analyzed amino acid scores across all datasets. Figure S7 in the Supplementary Material illustrates the distribution of these scores across sequence positions and residue types. The results indicate that *de novo* sequencing confidence remains consistently high across different peptide positions, with most residues achieving scores close to 1.0. This suggests that Casanovo reliably assigns amino acids with minimal uncertainty, even in complex bacterial datasets. We further found variations in amino acid confidence across different residue types, providing insights into peptide composition factors that sequencing reliability. These variations may be attributed to amino acid properties or fragmentation patterns. When MegaPX searches were applied to protein reference sequences, all peptides were correctly assigned to their respective target reference sequences (Supplementary Material Table S3). Our goal in this experiment is therefore not to reconfirm that these peptides are species-specific-this is already well established-but to assess whether a purely *de novo* workflow can recover and correctly assign such biomarkers in a realistic pathogenic context. In particular, the task is challenging because (i) *de novo* models may introduce local sequencing errors or truncate peptide termini, and (ii) closely related respiratory pathogens share substantial peptide sequence space, meaning that even small deviations from the canonical biomarker sequence could, in principle, lead to ambiguous or incorrect assignments.

Within this context, the 28 exact matches demonstrate that Casanovo attains sufficient residue-level accuracy for a substantial fraction of the proposed biomarkers, while MegaPX is able to map these *de novo* sequences back to the correct species in the presence of competing background proteomes. Moreover, our *k*-mer–based approximate string matching allows peptides with minor sequence deviations from the original biomarkers (e.g. single-residue substitutions or local shifts) to still be associated with the correct target proteome, rather than being discarded outright. This illustrates how *de novo* sequencing can support pathogen identification in situations where database-derived peptide lists may be incomplete or when sequence variation (strain diversity, mutations, resistance-associated changes) is expected.

We do not claim that species-unique peptide biomarkers cannot be detected by existing database-search or other identification workflows; when the exact peptide sequence is contained in the search space, such tools can reliably identify it. The added value of MegaPX in this experiment is that it can operate directly on *de novo* peptide predictions and still recover species-unique peptides with high specificity, without requiring pathogen-specific sequence databases. This suggests that *de novo*-driven MegaPX searches are viable for unique-peptide-based pathogen classification.

## Discussion

The development of MegaPX addresses significant challenges in metaproteomics, particularly those related to the taxonomic composition of microbiomes and the limitations of traditional database-dependent peptide identification methods. Our results demonstrate that not only is MegaPX efficient in handling large datasets but also significantly outperforms conventional tools like DIAMOND in both memory usage and hits assignment, particularly in scenarios involving large, complex databases. One of MegaPX’s key strengths is its ability to rapidly search massive databases like NCBI_nr (700+ million proteins) using only 0.177 GB of RAM, completing searches in ∼18 days. This highlights its scalability compared to traditional, resource-intensive methods. While high-performance computing could further reduce runtime via larger splits and parallelization, our focus remains on accurate classification using standard hardware. Performance on the Mix24X dataset further confirms MegaPX’s robustness in complex metaproteomic settings, accurately identifying short peptides with minimal overlap with conventional search results. When applied to the Lung Squamous Cell Carcinoma dataset, MegaPX showed promising results in the identification of human-related proteins, even in the presence of extensive post-translational modifications. Its high average protein matching rate of approximately 97% between the pseudo-ground truth and MegaPX underscores its reliability. Notably, MegaPX’s ability to handle mutation generation and still achieve accurate peptide assignment is particularly beneficial for cancer studies where mutations are frequent and can occur at any amino acid position. In the context of diagnostic applications, the identification of unique peptides across four bacterial species (Supplementary Material Table S3) highlights the potential of this approach for pathogen classification using mass spectrometry data. Notably, the high search engine scores (Supplementary Material Fig. S6) and consistent amino acid confidence levels (Supplementary Material Fig. S7) confirm the robustness the sequencing and assignment workflow. These findings highlight MegaPX’s capability to support rapid and reliable protein identification, which is essential for developing targeted diagnostic approaches.

MegaPX is a fast, memory-efficient, and accurate tool for peptide assignment and classification in metaproteomics. Its robust performance across diverse datasets, and adaptability to large search spaces, high mutation rates, and complex post-translational modifications, highlights its value for both research and clinical applications. As an effective preliminary screening tool, MegaPX provides tabular outputs that facilitate filtering based on peptide counts, enabling the creation of smaller, targeted proteome databases from large resources like NCBI_nr. These refined databases can enhance the accuracy and speed of downstream search algorithms. Future improvements in *de novo* sequencing also have the potential to improve our approach, as current algorithms are known to encounter issues in resolving sequence ambiguity, due to experimental properties such as missed cleavage sites or near-isobaric peptides (Beslic et al. 2023, van Puyenbroeck et al. 2025). Additionally, current de-novo models have a known bias towards species the model has seen during training (Hieu Tran et al. 2024). While imperfect, they generalize sufficiently to novel species to support downstream peptide assignment, and future architectures (e.g. transformer- or diffusion-based sequence models) are likely to further reduce such biases by learning more robust fragmentation patterns across taxa. Future improvements could further accelerate processing and extend MegaPX’s utility to other omics fields, such as proteogenomics. Integrating *de novo* peptide sequencing directly into MegaPX may offer additional benefits to users.

## Conclusion

This work demonstrates the effectiveness of *k*-mer-based taxonomy classification for fast and accurate indexing of amino acid sequences. Integrating *de novo* peptide sequencing enhances classification by reducing false positives often seen with approximate membership query (AMQ) structures. Incorporating mutated databases further improves peptide assignment, even in the presence of sequencing errors or disease-related mutations. Our optimizations highlight AMQ structures as a memory-efficient, accurate, and scalable solution for modern metaproteomics and bioinformatics applications.

## Supplementary Material

btag134_Supplementary_Data

## Data Availability

The data underlying this article are available in the article and in its online supplementary material.
