# Supplementary material for: MegaPX: fast and space-efficient peptide assignment method using IBF-based multi-indexing
Source: Bioinformatics. 2026 May 7;42(5):btag134. doi: 10.1093/bioinformatics/btag134 (PMC13148961; doi:10.1093/bioinformatics/btag134)
Supplement: btag134_Supplementary_Data [file btag134_supplementary_data.docx]

**Supplementary Materials for “MegaPX: fast and space-efficient peptide assignment method using IBF-based multi-indexing”**

**Ahmad Lutfi^1*^, Tanja Holstein^2,3,4^, Sandro Andreotti^5^, Thilo Muth^1*^**

**^1^** Data Competence Center MF2, Robert Koch Institute, Seestraße 10, 13353, Berlin, Germany

**^2^** VIB-UGent center for Medical Biotechnology, VIB, Belgium

**^3^** BioOrganic Mass Spectrometry Laboratory (LSMBO), IPHC UMR 7178, University of Strasbourg,

CNRS, Strasbourg, 67000, France

**^4^** Infrastructure Nationale de Prot´eomique ProFI FR2048, Strasbourg, 67087, France

**^5^** Department of Mathematics and Computer Science, Institute of Computer Science, Freie Universität Berlin, Takustr. 9, 14195, Berlin, Germany

**Supplementary Figures**


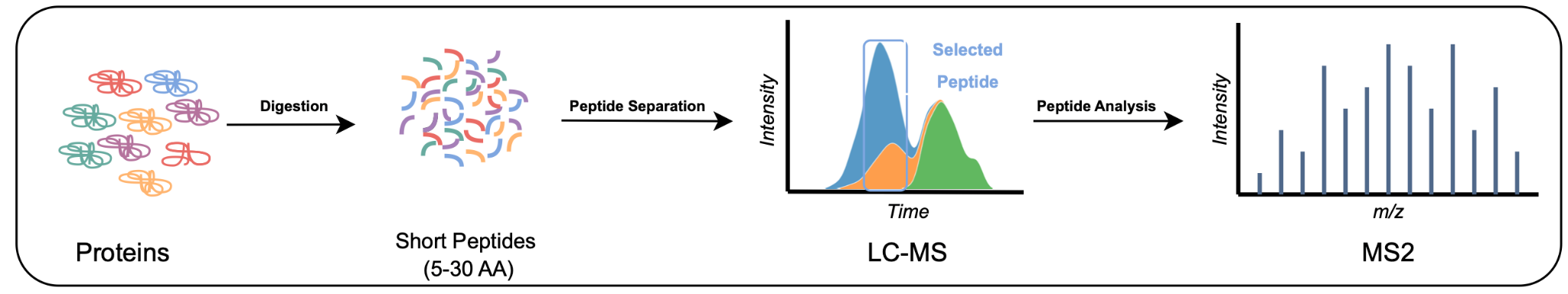


**Figure S1:** Bottom-Up Proteomics Workflow. Target proteins are digested by trypsin to cleave them into short peptides of length 5 to 30 AA. These peptides are then separated by LC and subsequently analyzed with the mass analyzer. The final MS/MS step describes the spectra generated for computer-aided analysis.


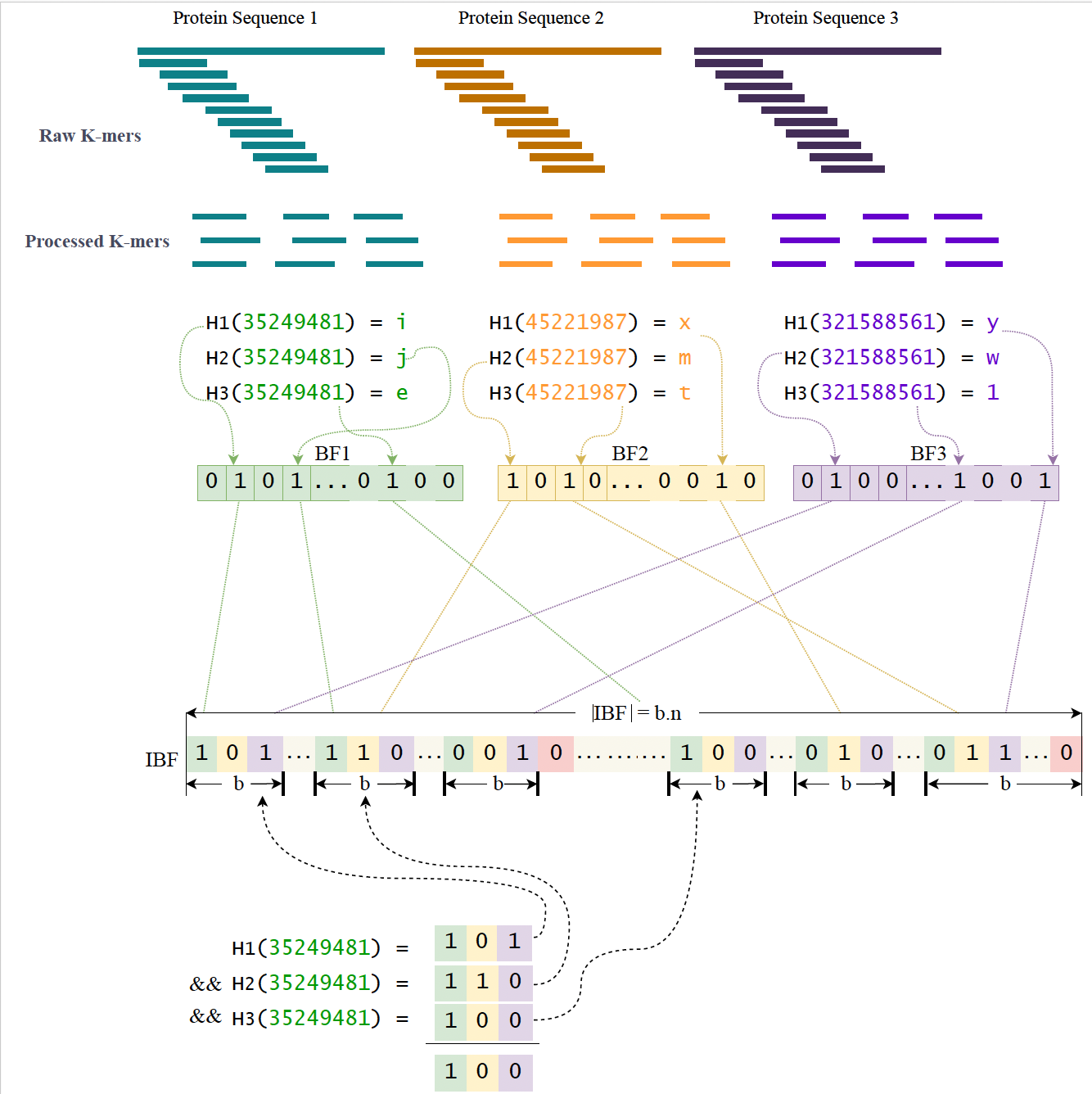


**Figure S2:** Construction and query of an Interleaved Bloom Filter (IBF).Raw peptide-derived k-mers are optionally processed (e.g., mutated) before insertion. Each bin corresponds to a separate protein or taxonomic unit and is color-coded consistently throughout the figure (Protein Sequence 1 = green, Sequence 2 = orange, Sequence 3 = purple). During insertion, each *k*-mer is hashed with three hash functions (H1-H3). For each hash value, the corresponding bit in the bin’s Bloom filter is set to 1. After the individual Bloom filters are constructed, they are interleaved into a single IBF composed of b sub-bit-vectors, one per bin, for each Bloom filter position. *The additional color appearing inside the IBF corresponds to more interleaved bit positions and does not represent a separate bin in this example*. During querying, the same hash functions are applied to a *k*-mer. The three retrieved sub-bit-vectors are combined with a bitwise AND operation (“&” in the figure). This operation identifies the bins for which all required bits are set indicating that the queried k-mer is present in those bins The IBF size is b·n, where n is the Bloom filter length per bin and b is the number of bins. When a new bin is added (i.e., a new reference sequence), the IBF expands to (b+1)·n because an additional sub-bit-vector must be allocated for the new bin.


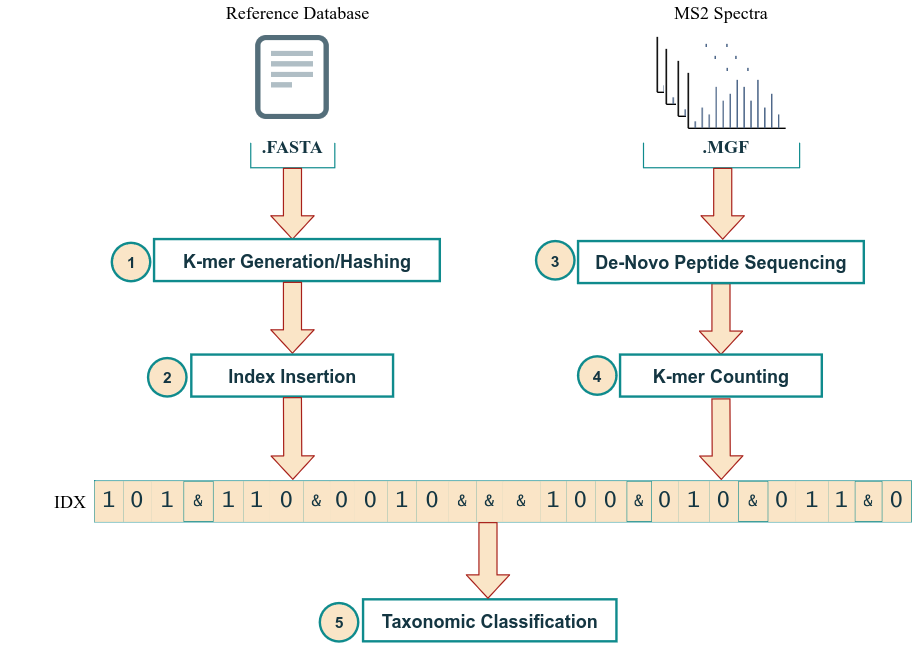


**Figure S3:** Classification Workflow. **(1)** Generate *k*-mers from each reference sequence and apply arithmetic coding with hashing to the *k*-mers. **(2)** Insert all generated *k*-mers into the corresponding index the user selects. **(3)** *de novo* peptide sequencing of the input spectra samples. **(4)** Counting the number of *k*-mers in the generated index. **(5)** Assign the counts and create a report on the classification of the last output of the taxonomy. The “&” symbols shown within the index are purely visual separators used to illustrate that the index is composed of multiple hashed bit positions; they do not represent a logical or mathematical operator in this figure.


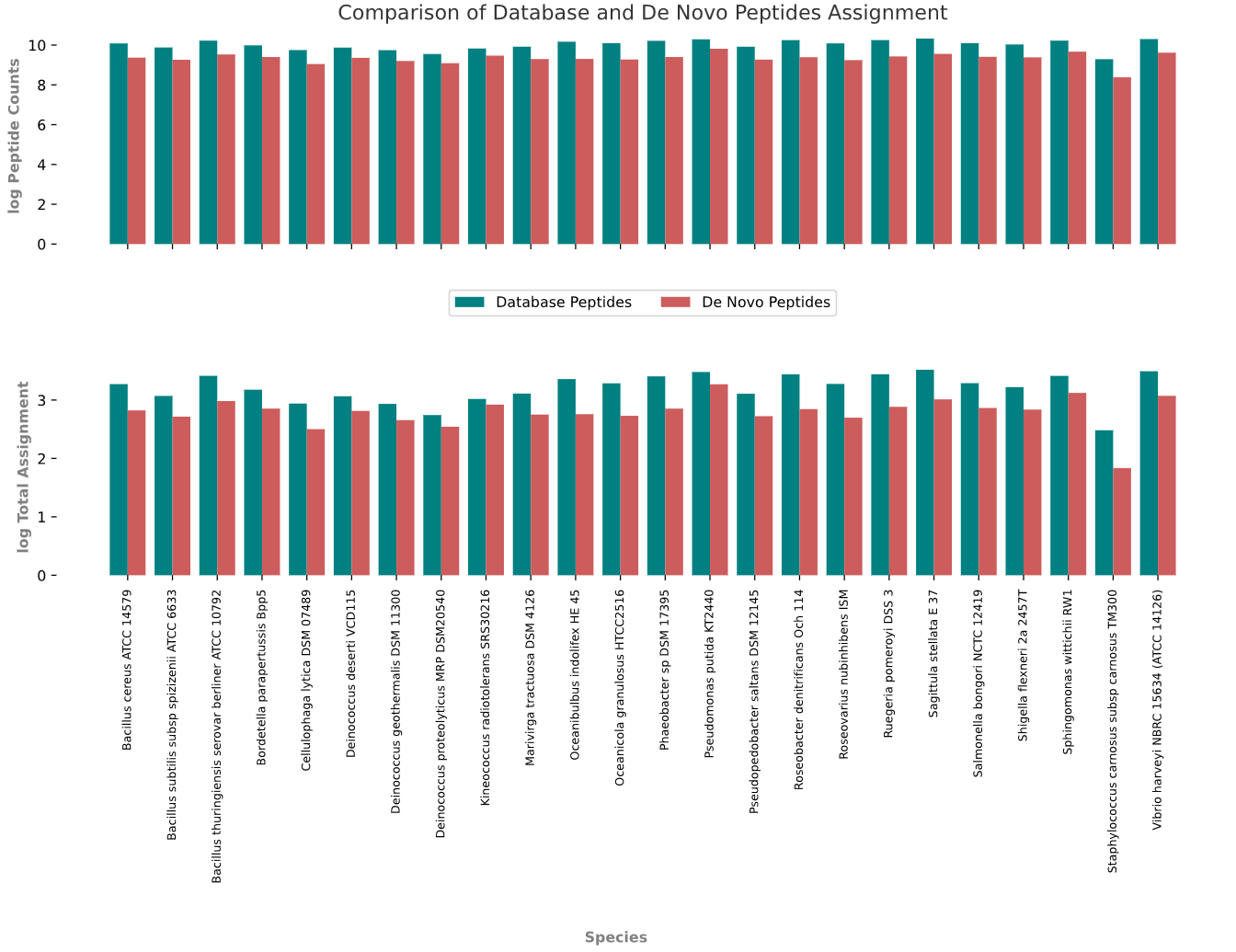


**Figure S4:** Species-level assignment overview for the Mix24X sample using database peptides and de novo peptides.The top panel shows, on a log10 scale, the number of peptides assigned to each species. Each peptide is counted once per species assignment.The bottom panel shows, on a log10 scale, the total number of species matches generated by each peptide set. Because individual peptides can match multiple species, this metric counts all species-level hits and therefore can exceed the peptide counts shown in the top panel.Assignments were computed with MegaPX using *k=5* and and complete peptide coverage, with minimizer precomputation disabled.


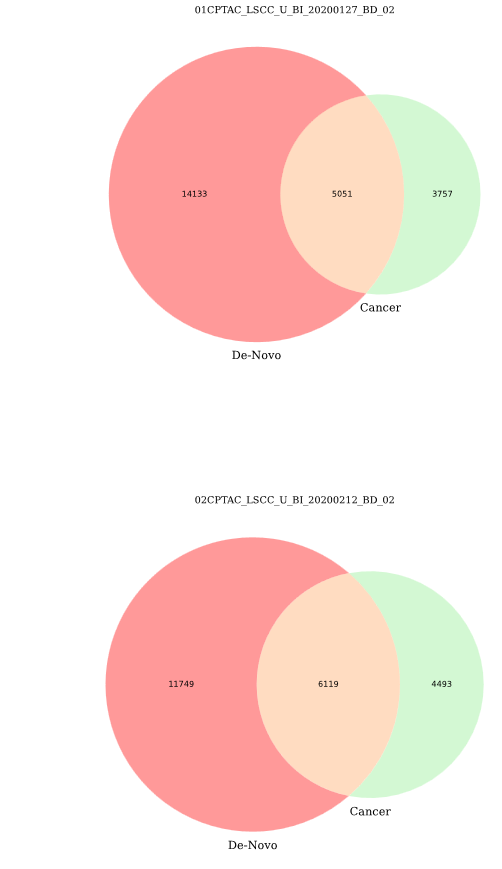


**Figure S5:** Set Membership of Shared Peptides in LSCC samples 01CPTAC_2 and 02CPTAC_2. This figure illustrates the overlap of shared peptides between *de novo* peptides and pseudo-ground truth peptides in LSCC samples 01CPTAC_2 and 02CPTAC_2, where *de novo* peptides have been filtered for duplications and a score threshold of 50%.


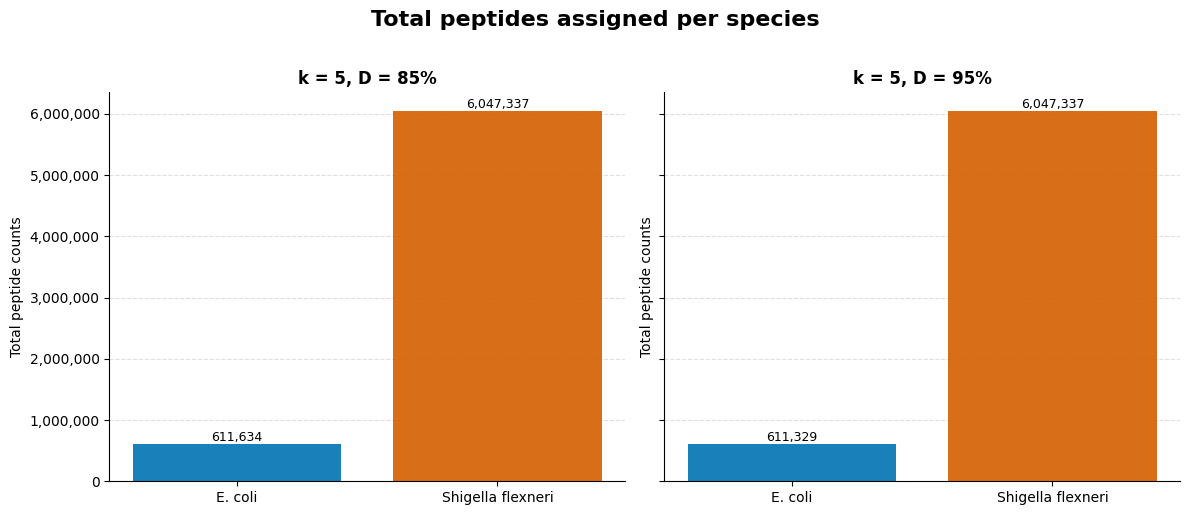


**Figure S6:** Proteome-level peptide assignments for E. coli and Shigella flexneri.Total numbers of peptides assigned to each species are shown for two parameter settings (l**eft:** *k=5,D=85%;* **right:** *k=5,D=95%*). In both cases, the vast majority of peptide assignments map to S. flexneri, while only a small fraction map to E. coli. This demonstrates that, when considering the entire proteome, the classifier strongly favors the true target species (S. flexneri) and that residual E. coli counts likely arise from shared peptide content between the two closely related genomes rather than from a dominant false-positive signal.


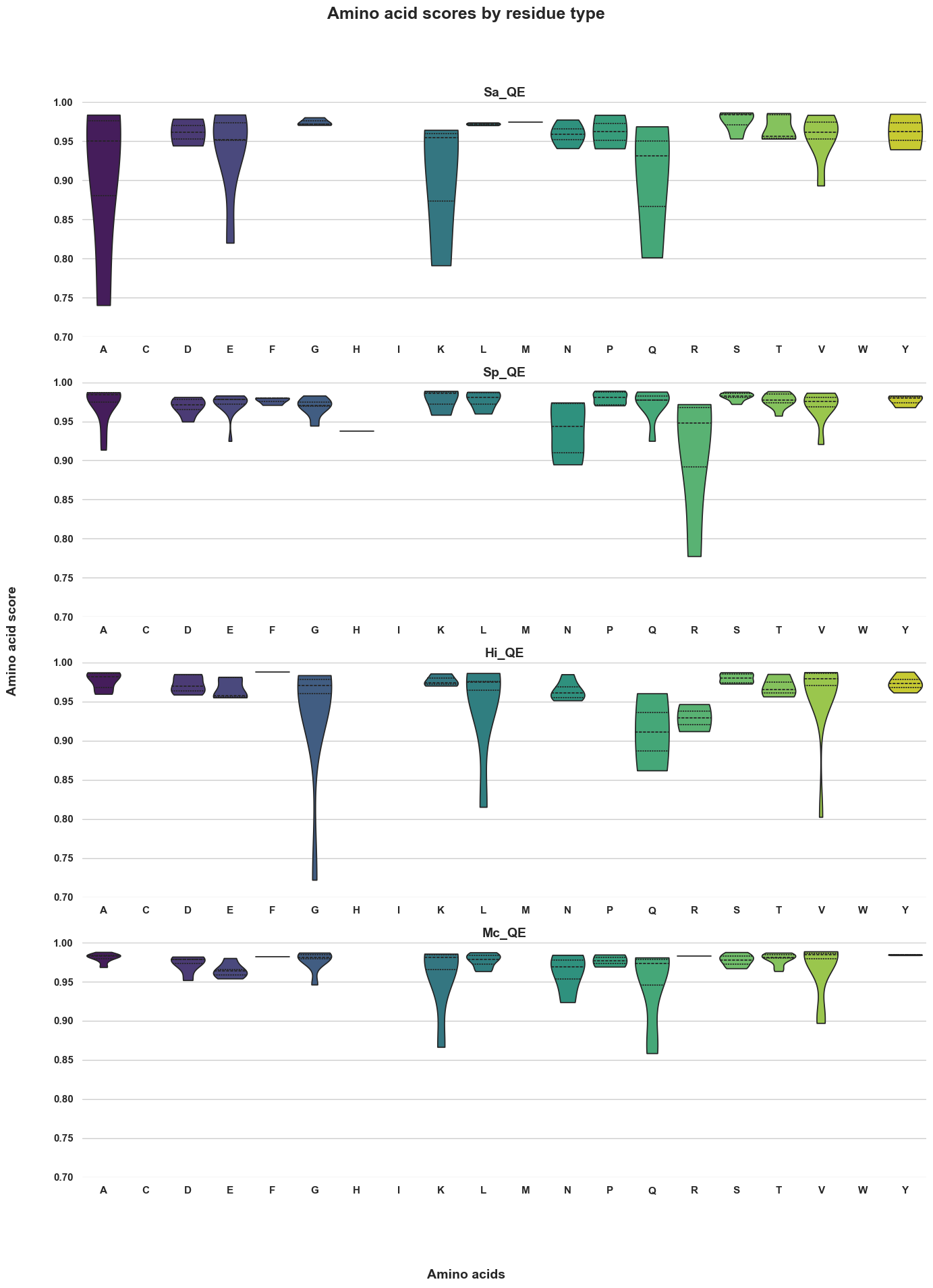


**Figure S7:** Distribution of amino acid-level confidence scores for identified peptide sequences across the four QE datasets (Sa_QE, Sp_QE, Hi_QE, Mc_QE). For each dataset, violin plots show the score distribution per residue type, summarizing both the central tendency and variability of the de novo sequencing scores. Overall, most residues exhibit high scores, indicating consistently high amino acid-evel confidence, with a few residue types showing broader distributions that reflect increased local sequencing uncertainty. *Sa_QE corresponds to Staphylococcus aureus, Sp_QE to Streptococcus pneumoniae, Hi_QE to Haemophilus influenzae, and Mc_QE to Moraxella catarrhalis.*


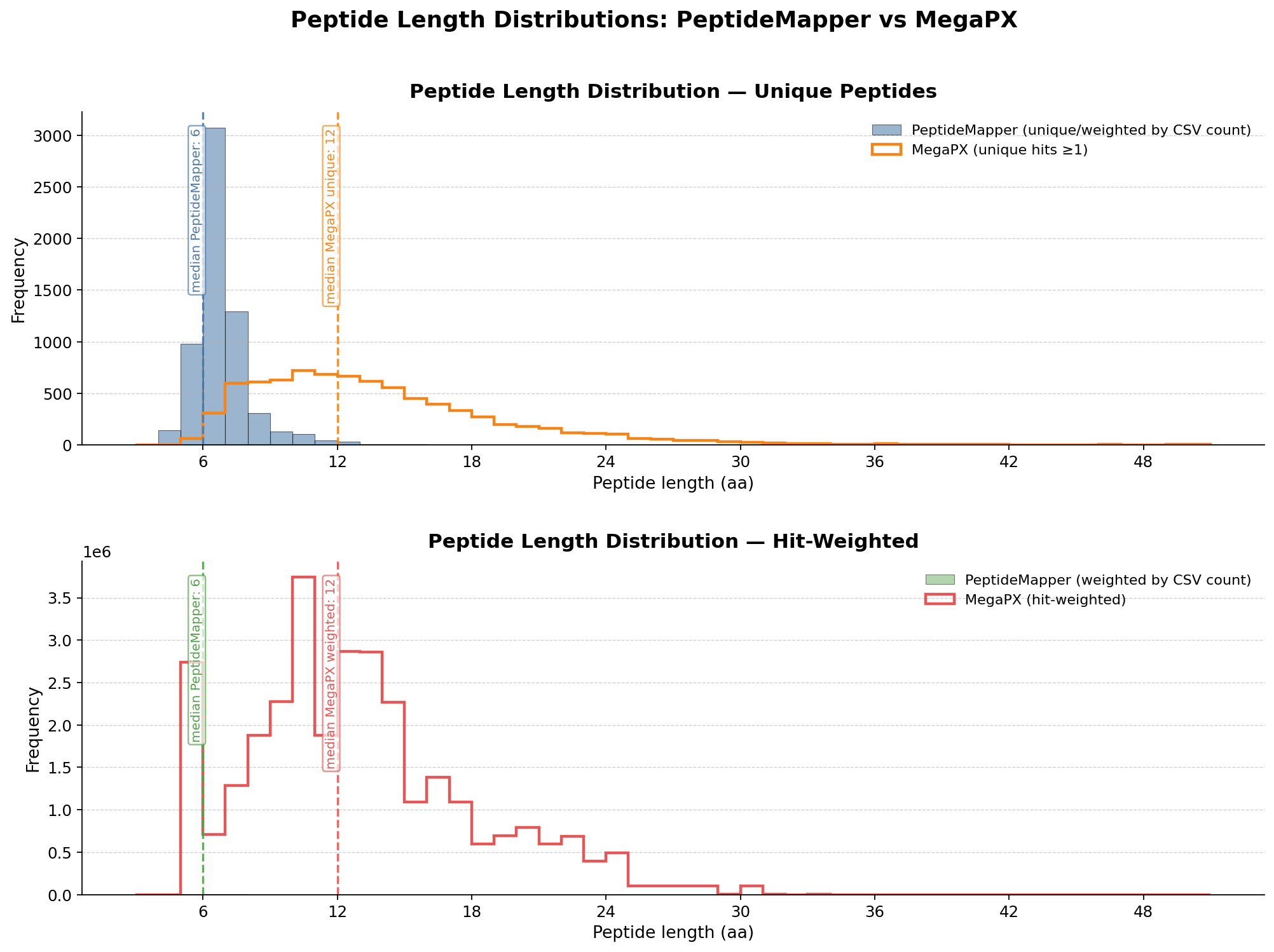
**Figure S8:** Peptide length distributions from PeptideMapper and MegaPX.Histogram comparison of peptide lengths using two views: **(top)** unique peptides-each distinct sequence counted once, regardless of how often it appears-and **(bottom)** hit-weighted counts-each peptide length repeated according to the number of matched references. Bins are 1 aa wide; dashed vertical lines mark medians. Across both views, MegaPX is shifted toward longer peptides and shows a higher median length. Longer peptides map more specifically and are less prone to spurious matches, so the MegaPX profile indicates greater mapping precision and reliability than PeptideMapper. In short: MegaPX is better because it concentrates evidence on longer, more discriminative sequences and maintains this advantage even when weighting by hits.

**Supplementary Tables**


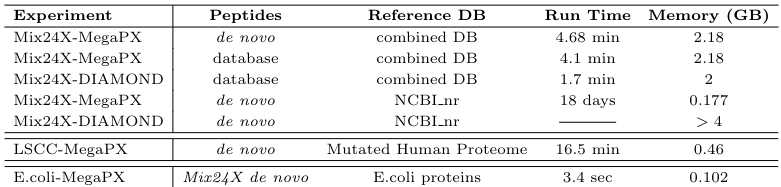


**Table S1:** Runtime and Memory Usage Analysis for Different Experiments. *Combined DB*: RefSeqBacterial proteins combined with the Mix24X targets.


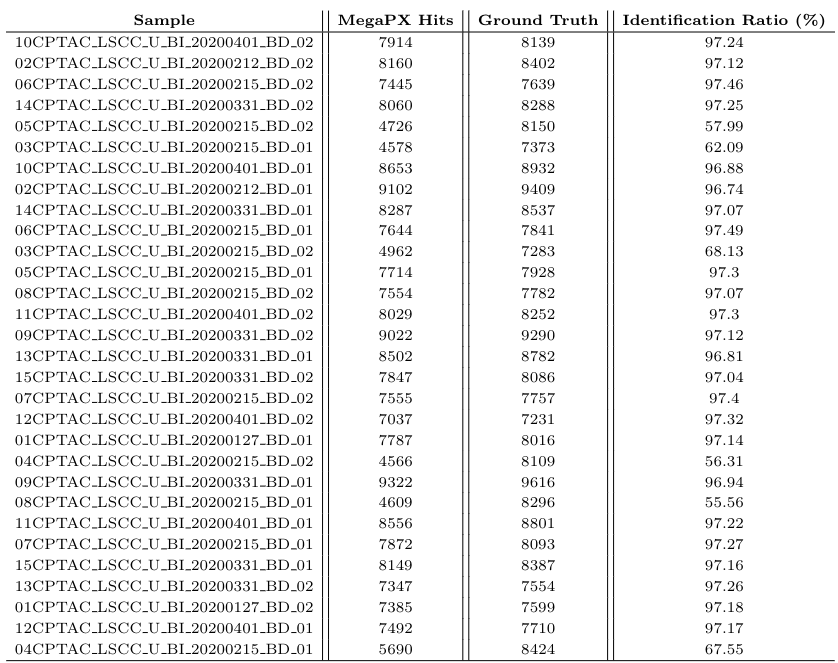


**Table S2:** Protein Matching Results Between MegaPX and Pseudo-Ground Truth Cancer Proteins. This table summarizes the number of proteins identified by MegaPX in each LSCC sample compared to the pseudo-ground truth cancer proteins. The matching ratio (%) represents the **recall**, calculated as the proportion of pseudo-ground truth proteins that were successfully identified by MegaPX. Specifically, it is computed by dividing the number of overlapping proteins by the total number of pseudo-ground truth proteins. While most samples achieve a high matching ratio (approximately 97%), a few outliers with lower ratios (57-68%) are noted.


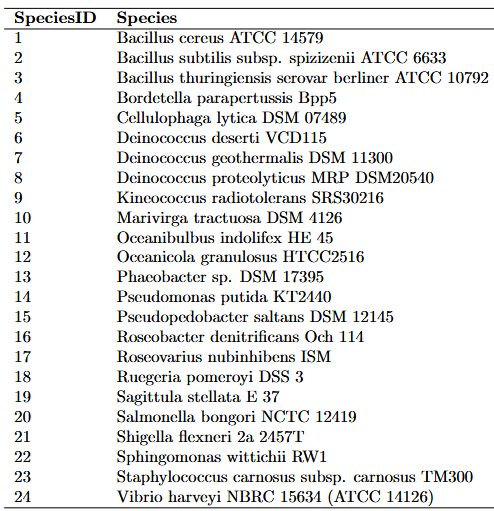


**Table S3:** Reference species included in the evaluation dataset using Mix24X dataset.


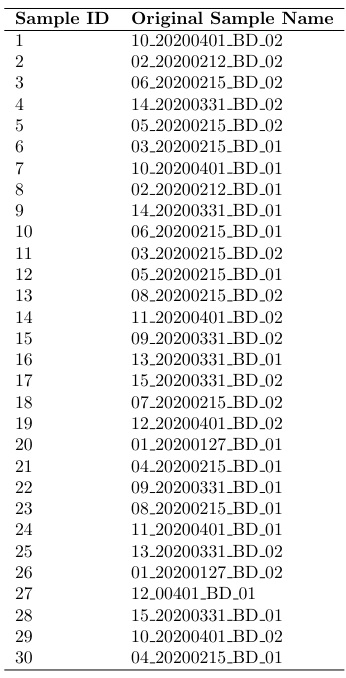


**Table S4:** Original sample name from the LSCC samples.


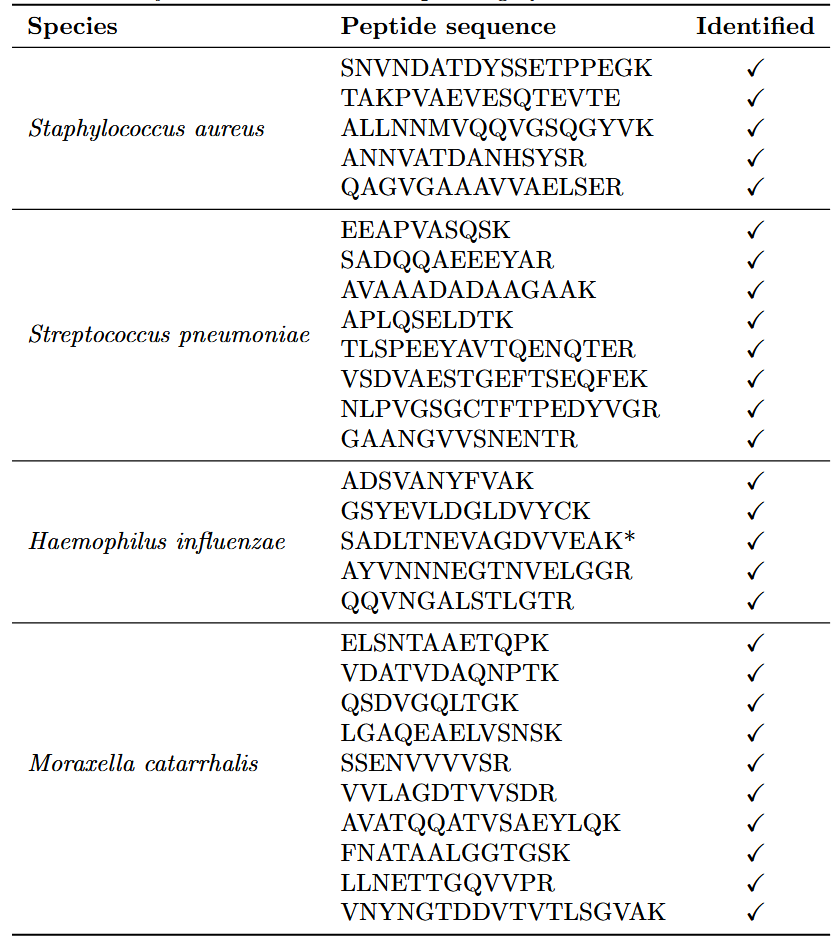


**Table S5:** De-novo identified peptide sequences for the four bacterial pathogens analyzed. For each species, all listed peptides were confidently identified in the corresponding dataset and are marked as detected in the *“Identified”* column.

**Supplementary Pseudo-Codes**


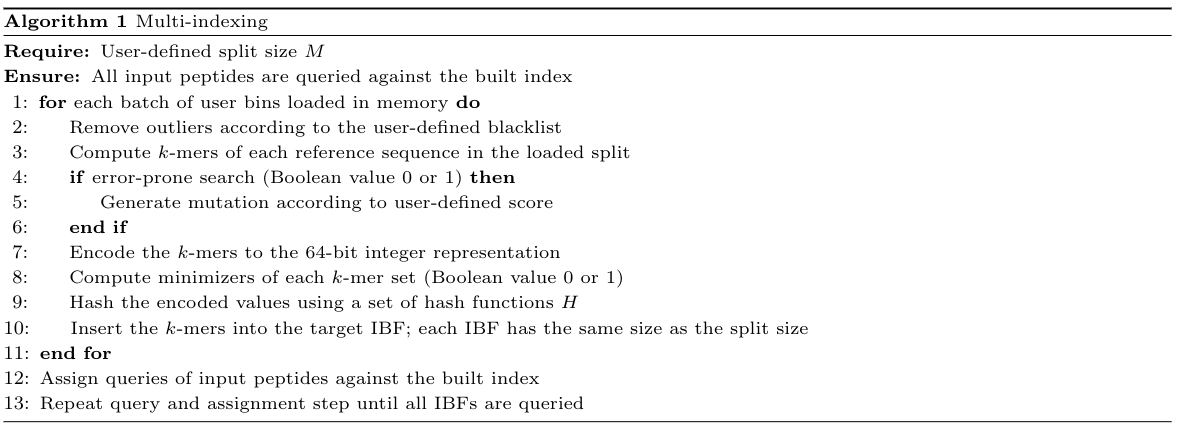


**Algorithm S1:** Multi-indexing Pseudo-code.

**Supplementary Experiments**

**Error-free classification**

The Mix24X metaproteomics sample contains MS bacterial datasets along with their corresponding references. Initially, these samples were sequenced using Casanovo and SMSNet, and the resulting peptides were pooled and filtered using a cutoff threshold 60%. These peptides were then searched against a combined database that included Mix24X target sequences and the NCBI RefSeqBacterial database (1,728,298 protein sequences). RefSeqBacterial, containing many bacterial protein sequences with a high similarity, was intended to simulate a realistic background for database searches in microbiome experiments. The combined database ultimately comprised around 2 million protein sequences. For our analysis and comparison with pseudo-ground truth peptides, we utilized the published identifications generated using Mascot Daemon software version 2.5.1 (Matrix Science). The peptide identifications, as described in the Mix24X publication, were performed with the *following parameters:* full trypsin specificity, allowance for up to one missed cleavage, static modification of carbamidomethylated cysteine (+57.0215), and variable oxidation of methionine (+15.9949). The mass tolerance was set to 5 ppm for parent ions, with MS/MS tolerances of 0.5 Da and 0.02 Da for the LTQ-Orbitrap XL and Q-Exactive HF instruments, respectively. Peptide matches with a Mascot peptide score corresponding to a p-value below 0.05 were retained. Protein identifications were considered valid if supported by at least two unique peptides. The false-positive rate for protein identification, estimated using a reverse decoy database search under the same parameters, was determined to be below 0.1%. To minimize false positives, a blacklist of overrepresented species was constructed, particularly those with many highly similar sequences from the same proteome. This reduces the number of non-target protein sequences that may otherwise appear frequently simply due to sequence redundancy or similarity. These species, characterized by a large number of proteins and amino acids irrelevant to our analysis, were excluded from the database. Examples include *Serratia* (18,717 proteins; 6,961,050 amino acids) and *Serratia nevei* (11,139 proteins; 4,489,936 amino acids). MegaPX searches were performed with a *k*-mer size of 5, split size of 1000, and full peptide set coverage (all *k*-mer should report hits to the protein sequences), with no minimizers applied. Proteins were concatenated into proteomes using a separator character (*) to prevent overlaps between sequences. This bulk concatenation approach significantly accelerates construction compared to inserting proteins individually into the IBF. In both cases, the IBF avoids inserting duplicate *k*-mers, as each *k*-mer is hashed and the corresponding bit positions are set.

We benchmarked DIAMOND and MegaPX under multiple search configurations to assess short-peptide alignment performance. DIAMOND was first run with its default parameters (e-value = 0.001, BLOSUM62, seed-and-extend), followed by NovoLign-style settings optimized for short peptides: PAM30, contiguous-seed mode, 85% identity, 80% query coverage, and --max-target-seqs 50. All tests used Mascot-identified peptides against the same target protein set, and e-value thresholds were varied from 0.001 to 10 to measure sensitivity inflation. MegaPX was run with its standard configuration (*k* = 5, three hash functions, and 0.80 coverage) for direct comparison.

**Error-tolerant classification**

The Lung Squamous Cell Carcinoma (LSCC) study includes multiple experiments and datasets designed for the proteomics analysis of LSCC. The primary objective of this analysis is to identify human-related proteins. The comprehensive dataset encompasses various MS-based analyses focusing on the phosphoproteome, acetylome, and ubiquitylome. Each sample was analyzed using a Thermo Fisher Scientific Orbitrap Fusion Lumos mass spectrometer equipped with a NanoSpray Flex NG ion source. Specifically, the ubiquitylome dataset, consisting of 30 MS/MS data sets, was analyzed and classified using MegaPX. The analysis of the original study aimed to identify proteins in 30 lung cancer samples. *De novo* peptide sequencing for these samples was conducted using both Casanovo and SMSNet resulting in 608,710 peptide suggestions. Following the *de novo* sequencing, peptides with scores below a 50\% threshold were filtered out to ensure data quality. The study includes pseudo-ground truth peptides determined from a database search using MS-GF+ v2017.01.27. Peptide-spectrum matches were identified based on the following database search parameters: a 20 ppm precursor mass tolerance (*-t 20ppm*), semi-tryptic digestion (*-ntt 1*), and a target-decoy approach (*-tda 1*) for FDR estimation. The instrument type was set to Orbitrap (*-inst 1*), and fragmentation mode was specified as HCD for Q-Exactive (*-m 3*) or CID for Orbitrap (*-m 1*). The search allowed up to one isotope error (*-ti 0,1*) and considered peptides up to 50 amino acids in length (*-maxLength 50*). For the LSCC experiments, SMSNet and Casanovo de novo peptides were filtered for duplicates and required to meet a 50% confidence score. These peptides were searched against the human RefSeq protein database using MegaPX in mutation mode with *k* = 5, mutation score = 25, and a full assignment threshold (99%). All reference proteins were converted into mutated *k*-mers under these settings. Searches were run using 114 IBFs.

**Evaluation of diagnostic applications**

***Sample search against highly similar proteomes***

We searched the Mix24X peptides against the complete protein database of *E.coli* (strain K12) and *Shigella flexneri*, both downloaded from UniProt. The dataset contains a total of 6,460 proteins in *E.coli* and 63,867 in *Shigella flexneri*. Peptides were searched using a *k*-mer size of 5 and assignment thresholds of 85% and 95%. The search was performed without mutation generation, as gap-based searches were not required. MegaPX utilized a split size of 1,000 user bins per run.

***Species-unique peptides***

In another experiment, we applied our approach to real bacterial pathogens. In the corresponding publication, species-unique peptide biomarkers for respiratory tract pathogens including *Streptococcus* *pneumoniae*, *Haemophilus* *influenzae*, *Moraxella catarrhalis*, and *Staphylococcus aureus* were identified. The study highlighted the most promising candidate peptide biomarkers for these four species. In our experiment, we sequenced the corresponding MS datasets (data are available via ProteomeXchange with the identifier PXD014522) using Casanovo and then searched the identified peptide biomarkers within the target 50-reference proteins. This served two purposes: first, to demonstrate that Casanovo can accurately sequence unique peptides in a pathogenic scenario, and second, to show that MegaPX, utilizing the *k*-mer approach (*k=5*), can correctly identify the target reference protein based on these unique peptides. For this experiment, we specifically searched for unique peptides to assess the accuracy of approximate string matching in classifying species-specific unique sequences; we aimed to demonstrate that approximate string matching can reliably classify peptides as species-specific while also being capable of correctly assigning exact matches when no mutations or substitutions are present. This highlights the method's ability to balance flexibility with precision in sequence classification.
